# Postprocessing of Structural En Face OCT Images of the Choroidal Haller Layer for Quantitative Assessment: An Exploratory Pilot Study

**DOI:** 10.3390/biomedicines14102351

**Published:** 2026-10-15

**Authors:** Anait S. Khalatyan, Yusef Yusef, Konstantin S. Avetisov, Sergey A. Smolin, Vladimir I. Siplivy, Anna E. Aslamazova, Ludmila V. Reznikova, Liubov V. Machekhina, Alexandra A. Melnitskaia, Irina D. Strazhesko

**Affiliations:** 1 Krasnov Research Institute of Eye Diseases, Moscow 119021, Russia; info@eyeacademy.ru (Y.Y.); avetisov.k.s@gmail.com (K.S.A.); smolin_s_a@staff.sechenov.ru (S.A.S.); 2 Department of Ophthalmology, Sechenov First Moscow State Medical University ( Sechenov University), Moscow 119991, Russia; siplivyy_v_i@staff.sechenov.ru (V.I.S.); aslamazova_a_e@staff.sechenov.ru (A.E.A.); reznikova_l_v@staff.sechenov.ru (L.V.R.); 3 Russian Gerontology Research and Clinical Centre, Pirogov National Research Medical University, Moscow 129226, Russia; machehina_lv@rgnkc.ru (L.V.M.); melnickaya_aa@rgnkc.ru (A.A.M.); strazhesko_id@rgnkc.ru (I.D.S.)

**Keywords:** aging, choroid, Haller layer, swept-source optical coherence tomography, structural en face imaging, Phansalkar thresholding, image postprocessing

## Abstract

**Background/Objectives**: To develop a binarization workflow for structural en face OCT images of the choroidal Haller layer and examine how postprocessing parameters affect measurements, using chronological age as an exploratory reference. **Methods**: We included 66 healthy volunteers (66 eyes; 22–73 years; three age groups). En face Haller layer images from swept-source OCT angiography scans (DRI OCT Triton; 6 × 6 mm) were binarized using retinal vessel masking and Phansalkar thresholding. Radius R (22 values) and coefficients k and r (24 combinations) were varied. **Results**: Measurements depended strongly on R; age-group separation was similar over R = 13–26 pixels; permutation testing gave *p* = 0.031 across radii and *p* = 0.115 across all 45 configurations. Using the representative working configurations (R = 17 pixels; default k = 0.25 and r = 0.50), vessel density (VD), vessel length density (VLD), and inter-vessel distance (IVD) showed modest, non-monotonic nominal age-group differences (η^2^ ≈ 0.10); those for VD and IVD were lost after accounting for masked regions. Adjusted for sex and spherical equivalent, VLD decreased and IVD increased with age, whereas VD did not. **Conclusions**: Measurements depend substantially on postprocessing parameters. The working configuration, selected and evaluated in one cohort, requires repeatability testing and independent validation.

## 1. Introduction

The Haller layer comprises large choroidal vessels, approximately 100–300 µm in diameter, between the Sattler layer and the suprachoroidal space or lamina fusca of the sclera [1,2,3,4,5]. Its transition to the Sattler layer is not sharply defined [1].

Enhanced-depth and swept-source OCT allows visualization of the choroid to the sclerochoroidal junction. Medium and large vessels appear as hyporeflective lumina surrounded by more reflective tissue [1,4].

The advent of OCT angiography (OCTA) has substantially expanded the possibilities of noninvasive choroidal imaging. This technology delineates vessel contours from the decorrelation signal of moving erythrocytes; however, reliable flow detection is achievable mainly at the level of the choriocapillaris. When segmentation is performed at the level of the Sattler and Haller layers, the vessels are visualized chiefly as projection artifacts of their lumina. Notably, clearer visualization of large-caliber choroidal vessels is possible on en face images, which represent a reconstruction of near-infrared reflectance signal intensity averaged within a depth-defined tissue slab. Because the vessels of the Haller layer are visualized most distinctly on en face images, quantitative vascular assessment is most often performed precisely for this layer [6,7,8].

Reported structural choroidal measurements include vessel area and total vessel length [6,7,8], vessel density [7,9,10], vessel length density [11], vessel diameter [6,7,12], and the number of vascular intersections [7,8]. Inter-vessel distance has also been measured manually between selected large vessels on three-dimensional choroidal maps [13].

Binarization must account for the larger caliber of Haller vessels compared with the choriocapillaris (up to 300 versus approximately 20 µm) [1,2]. These features affect the local threshold window and subsequent morphological processing.

Published choroidal binarization approaches include Otsu [9], Niblack [10], Bernsen [14], and Phansalkar [15]. Differences in image type, slab selection, and threshold settings limit direct comparison of quantitative measurements and motivate explicit reporting and systematic evaluation of processing parameters.

A problem common to both OCT angiograms and en face images is the presence of projection artifacts from the overlying large retinal vessels, which, without dedicated processing, are erroneously interpreted by the binarization algorithm as choroidal vessels and lead to systematic overestimation of certain parameters. To mitigate this limitation, the device software incorporates a projection artifact removal (PAR) algorithm for OCT angiograms [16,17]; however, even this algorithm cannot always fully eliminate the error, and it is not applied to en face images.

Age-related choroidal thinning [18] and disease-associated vascular remodeling [19,20] provide a biological context for these measurements. Refraction is also relevant: myopia is associated with choroidal thinning [21,22], and high myopia with reduced Haller vessel density and an inverse association with axial length [23]; hyperopia is associated with choroidal thickening [10,24].

This exploratory study aimed to develop a binarization workflow for structural en face images of the Haller layer, to examine the sensitivity of quantitative measurements to Phansalkar parameters, and to define a representative working configuration using chronological age as an exploratory reference.

## 2. Materials and Methods

### 2.1. Study Design, Ethics, and Participants

This prospective observational pilot study included 66 healthy volunteers (66 eyes), aged 22–73 years. The local ethics committee approved the protocol (No. 59, 13 September 2022), and all participants provided written informed consent in accordance with the Declaration of Helsinki. Eligibility required age 20–90 years and capacity to consent. Exclusion criteria were high refractive error, best-corrected visual acuity below 0.8, media opacity, retinal or choroidal disease, systemic disease affecting choroidal blood flow, or motion artifacts. Refraction was measured by autorefractometry after a single instillation of tropicamide 0.8%/phenylephrine 5% (Fenikamid, Grotex, Russia); the spherical equivalent was calculated as the sphere plus half the cylinder. The spherical equivalent of the included eyes ranged from −3.0 to +3.0 D (Table 1).

### 2.2. Imaging Protocol and Image Export

Macular OCTA scans (6 × 6 mm) were acquired using swept-source DRI OCT Triton (Topcon, Tokyo, Japan; wavelength 1050 nm). OCTA scans were acquired before pupil dilation. Scans were repeated until a Q-index of at least 95 was obtained, and the first scan meeting this criterion was analyzed. One eye per participant was analyzed: the right eye by default, or the left eye if this quality could not be achieved for the right eye.

**Figure 1:**
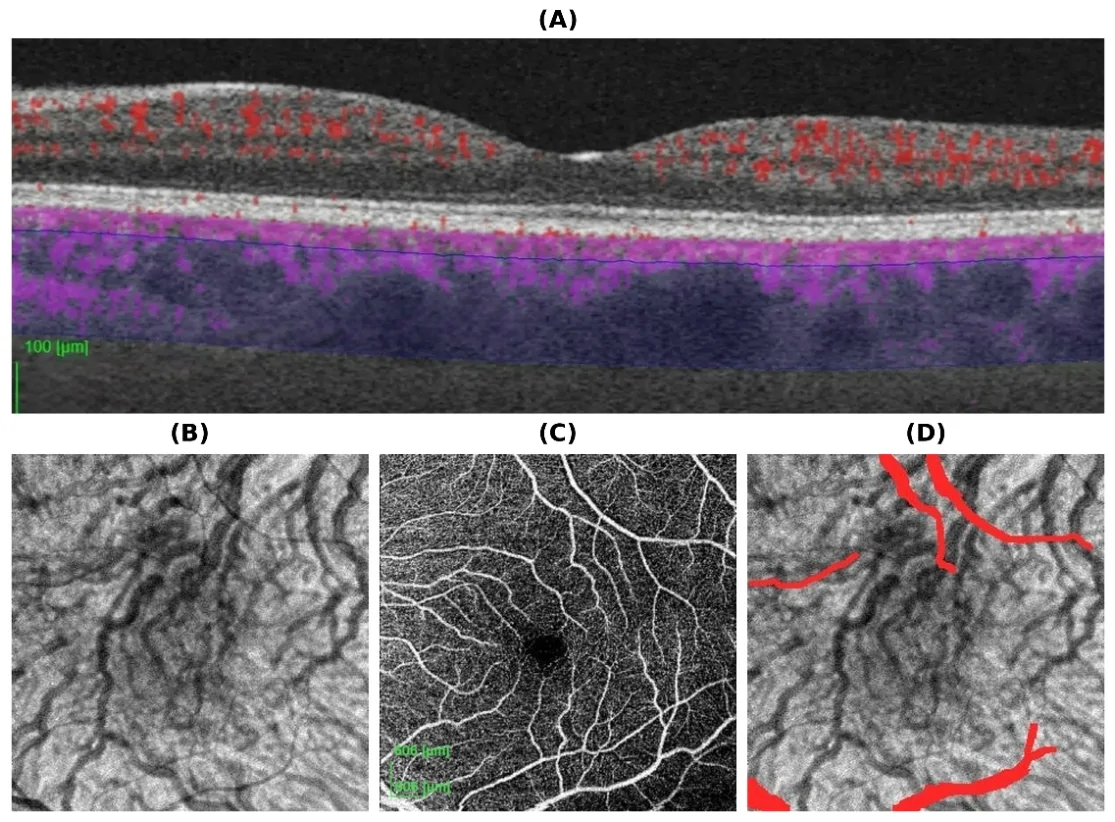
Structural OCT, en face imaging, and artifact masking. (**A**) Structural OCT B-scan with flow overlay and Haller layer segmentation; the original 100 µm scale bar is retained. (**B**) Haller layer en face projection. (**C**) Superficial retinal plexus OCT angiogram from the same scan. (**D**) Retinal vessel exclusion mask overlaid in red on panel B. Panels B–D represent a 6 × 6 mm field; the native en face exports were 320 × 320 pixels.

The Haller layer was segmented manually in IMAGEnet 6 by one investigator (S.A.S.), masked to participant age and age group, because automatic segmentation was unavailable. The upper and lower slab boundaries followed the corresponding vessel margins on structural OCT B-scans (Figure 1A).

En face images of this slab, generated in IMAGEnet 6 as the mean OCT reflectance intensity within the slab (Enface Type: Average; Figure 1B), and superficial retinal plexus OCT angiograms from the same scan (Figure 1C) were exported as uncompressed 8-bit TIFF images (320 × 320 pixels).

The native spatial scale was 18.75 µm per pixel.

Images were analyzed in Fiji (v. 1.54p; National Institutes of Health, Bethesda, MD, USA) using an in-house macro, with spatial calibration applied through Set Scale.

### 2.3. Image Postprocessing and Parameter Calculation

**Figure 2:**
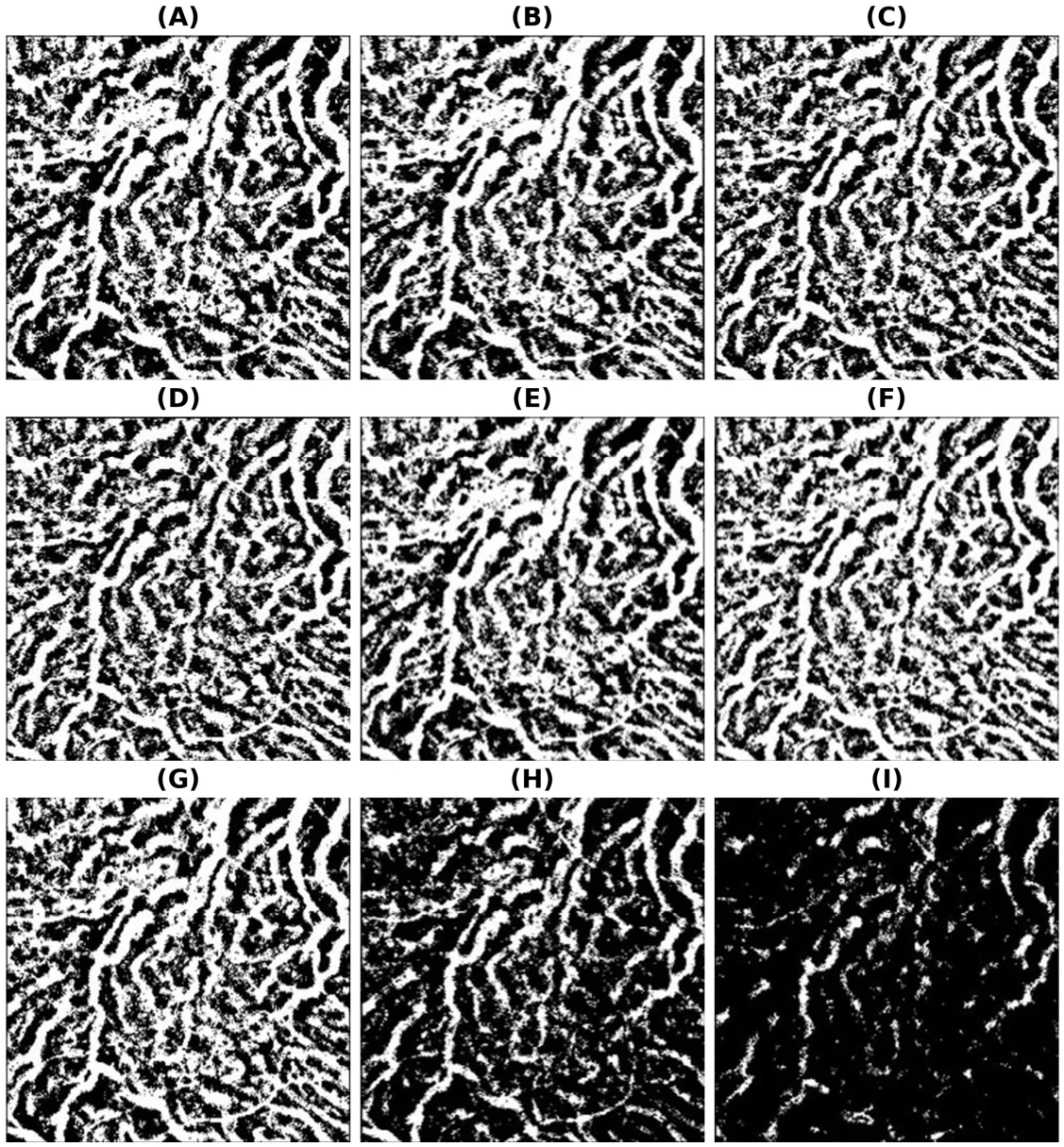
Local binarization of a representative unfiltered Haller layer en face image using the default settings of Fiji’s Auto Local Threshold module (R = 15 pixels). (**A**) Bernsen; (**B**) Contrast; (**C**) Mean; (**D**) Median; (**E**) MidGrey; (**F**) Niblack; (**G**) Otsu; (**H**) Phansalkar; (**I**) Sauvola.

Nine algorithms in Fiji’s Auto Local Threshold module (Bernsen, Contrast, Mean, Median, MidGrey, Niblack, Otsu, Phansalkar, and Sauvola; Figure 2A–I) were compared on Haller layer en face images from 30 randomly selected eyes (10 per age group), using the default parameters of each method at a fixed local radius of 15 pixels (approximately 281 µm). The comparison was performed on unfiltered images, without FFT bandpass filtering. Two researchers jointly assessed the binarized images against the corresponding original images, considering preservation of visible vessel contours, separation of adjacent vessels, vessel loss, and false inclusion of background regions; the assessors were not masked to algorithm identity but were masked to participant age group.

At the default local radius of 15 pixels, Bernsen, Contrast, Mean, Median, MidGrey, Niblack, and Otsu produced dense masks in which adjacent vessels merged and background regions were included (Figure 2A–G), whereas Sauvola produced a sparser mask with the loss of peripheral vessel pixels (Figure 2I). In this qualitative comparison, Phansalkar appeared to separate adjacent vessels best while preserving visible vessel contours (Figure 2H) and was therefore selected for parameter evaluation.

For all subsequent analyses, Haller layer images underwent FFT bandpass filtering before Phansalkar thresholding with large- and small-structure settings of 40 and 3 pixels, respectively, to reduce background heterogeneity and fine-scale noise.

To exclude retinal vessel projections from Haller layer measurements, a mask was automatically generated from the superficial capillary plexus (SCP) angiogram. Rolling-background subtraction (50 pixels) and Gaussian smoothing (σ = 1 pixel) were applied to reduce background variation and noise, followed by binarization using a fixed intensity threshold of 160–255.

The binary mask was refined by morphological closing (one dilation followed by one erosion with a 3 × 3-pixel square neighborhood) and removal of objects smaller than 150 pixels (0.053 mm^2^). The mask was then dilated by two iterations, extending it by 2 pixels (37.5 µm) beyond vessel boundaries; within a 38-pixel (approximately 0.71 mm; 12% of the image width) band along each image edge, two additional iterations extended it by 4 pixels (75 µm) in total to cover residual retinal vessel projections (Figure 1D).

SCP masking was applied to all images. The exclusion mask was subtracted from the binary Haller vessel mask after Phansalkar thresholding and morphological opening, but before local-thickness filtering.

The Phansalkar threshold is *T* = *m* × [1 + *p* × *exp*(−*q*·*m*) + *k* × (*s*/*r* − 1)], where m and s are the local intensity mean and standard deviation within radius R; k weights the local contrast, r normalizes it, and *p* = 2 and q = 10 are fixed constants [25].

Phansalkar thresholding was applied without the “white objects on black background” option, so that pixels at or below the local threshold, corresponding to hyporeflective vessel lumina, were set to white (255); no intensity inversion was applied before thresholding.

In RSWEEP mode, R varied from 5 to 26 pixels (93.75–487.50 µm), with k = 0.25 and r = 0.50 fixed, yielding 22 configurations.

In krSWEEP mode, R was fixed at a representative working radius of 17 pixels while k (0.10, 0.15, 0.20, 0.25, 0.30, 0.35, 0.40, 0.50) and r (0.25, 0.50, 0.75) were varied, yielding 24 configurations.

In total, 45 unique configurations were evaluated, because the configuration R = 17 pixels, k = 0.25, r = 0.50 was included in both modes.

**Figure 3:**
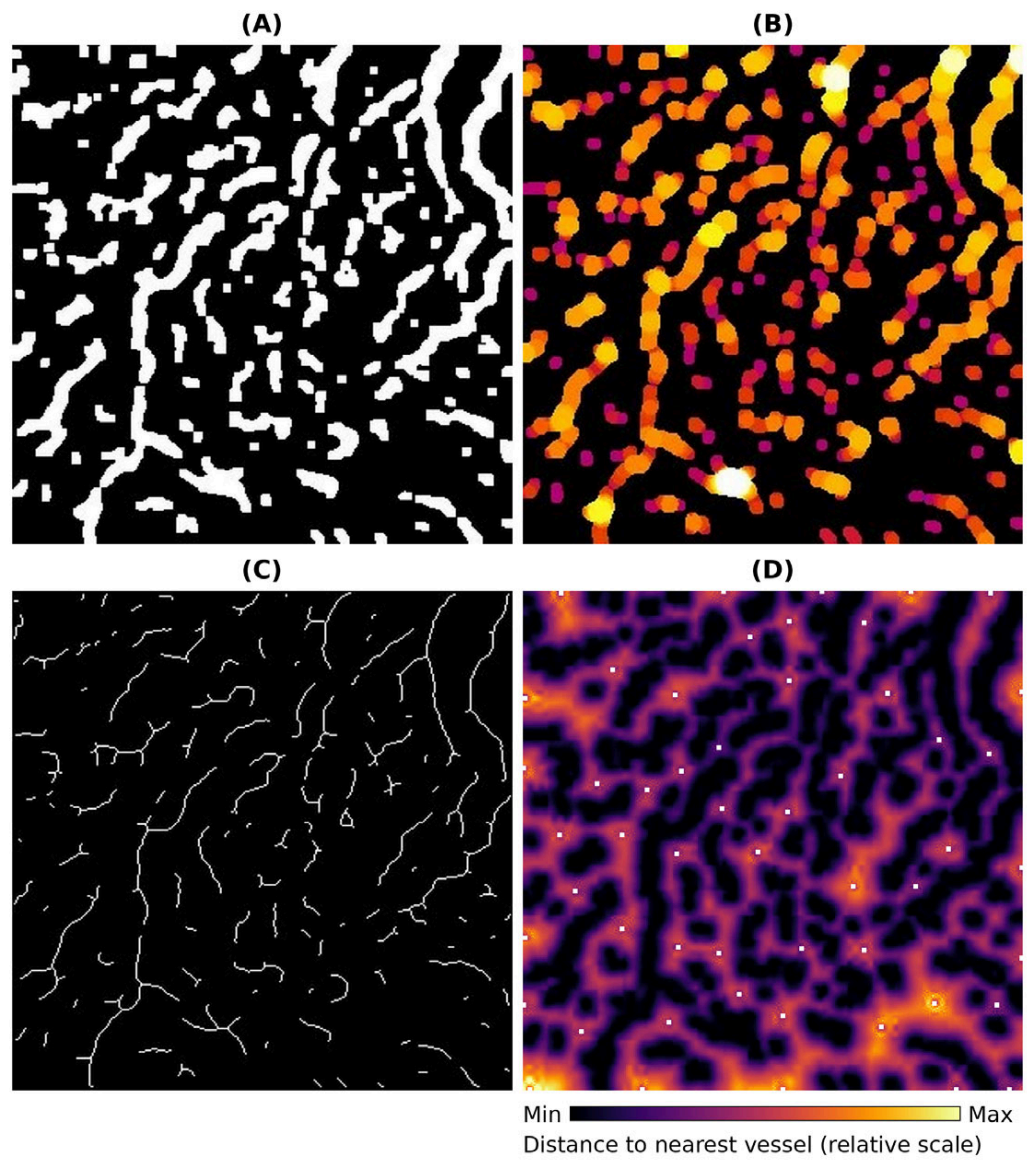
Postprocessing and distance mapping. (**A**) Binary Haller vessel mask at R = 17 pixels. (**B**) Local-thickness map for diameter-based assessment and filtering. (**C**) Vascular skeleton. (**D**) Euclidean distance-to-nearest-vessel map, with brighter colors indicating larger distances and white points marking local maxima. Colors in (**B**,**D**) are scaled to the range of each map and indicate relative values only.

Postprocessing of the Haller vessel mask included morphological opening consisting of two erosions followed by two dilations with a 3 × 3-pixel square neighborhood, which removed foreground regions that could not contain a 5 × 5-pixel square (approximately 94 × 94 µm); no minimum object size was applied. After subtraction of the SCP exclusion mask, local-thickness filtering removed vessel pixels whose local thickness, defined as the diameter of the largest inscribed circle containing the pixel, was below 80 µm (approximately 4.3 pixels); pixels at or above this threshold were retained. The filter acted on pixels rather than on whole objects, so thin portions of otherwise retained vessels were also removed (Figure 3A,B). Because opening had already removed narrower structures, this filter mainly affected thin remnants left along the edges of the SCP exclusion mask.

The binary mask was skeletonized, and branch lengths were measured using AnalyzeSkeleton (Figure 3C). VD was calculated as the percentage of vascular pixels, VesselArea as their area in mm^2^, and VLD as total skeleton branch length per unit area. VD and VLD were normalized to the full 36 mm^2^ field, including SCP-excluded regions. In a sensitivity analysis, VD and VLD were also normalized to the analyzable area (36 mm^2^ minus the SCP exclusion mask). Full-field normalization was retained as the primary analysis so that all eyes shared the same denominator; because masked regions cannot contain segmented Haller vessels, it may underestimate VD and VLD in eyes with larger masks.

MeanDiameter was the mean nonzero local thickness, expressed in micrometers, and VesselCount was the number of connected binary objects in the final mask.

IVD was calculated as twice the mean value at local maxima of the Euclidean distance map of the nonvascular background, converted to micrometers (Figure 3D). Local maxima were detected with a prominence of 3 pixels (1 pixel if none were detected); maxima on the image border were not excluded. It characterizes intervascular space size rather than center-to-center vessel distance; SCP-excluded regions were included in the background. In a sensitivity analysis, IVD was recalculated after excluding maxima whose inscribed circle overlapped the SCP exclusion mask, so that only intervascular spaces bounded by segmented Haller vessels were retained.

### 2.4. Statistical Analysis

Statistical analyses used IBM SPSS Statistics 26.0 (IBM Corp., Armonk, NY, USA); bootstrap, permutation, and regression analyses were performed in Python 3 (SciPy and statsmodels libraries). Data are presented as mean ± standard deviation. Normality and variance homogeneity were assessed using Shapiro–Wilk and Levene’s tests. Each processing configuration was analyzed separately for the same 66 eyes of 66 participants. The sex distribution across age groups was compared using Fisher’s exact test, and spherical equivalent by one-way ANOVA.

Age groups were compared by one-way analysis of variance (ANOVA), with η^2^ as the effect size and Bonferroni correction for the three pairwise comparisons. Confidence intervals (CIs) for η^2^ were obtained by age-stratified bootstrap resampling (5000 replicates), and CIs for pairwise mean differences were Bonferroni-adjusted. When ANOVA assumptions were not met, Welch ANOVA and the Kruskal–Wallis test were also applied. In sensitivity analyses, age-group comparisons were adjusted for sex and spherical equivalent (analysis of covariance), and each parameter was regressed on age as a continuous variable with sex and spherical equivalent as covariates in all participants and in women only; a quadratic age term was tested to assess non-linearity.

Both sweeps (RSWEEP and krSWEEP) were evaluated using the same criterion, which was the summed effect size (Ση^2^), defined as the sum of age-group η^2^ values across five parameters: VD, VLD, MeanDiameter, VesselCount, and IVD. VesselArea was not included because, for a fixed field area, it duplicates VD. As a sensitivity check applied to both sweeps, Ση^2^ was also calculated across four parameters, excluding VesselCount, because the number of objects depends on mask fragmentation. A plateau was defined as the range of configurations with Ση^2^ ≥ 90% of the maximum within a sweep. Uncertainty was assessed by age-stratified bootstrap resampling of eyes (5000 replicates, with the same resampled eyes for all configurations), with percentile 95% CIs for paired differences in Ση^2^ relative to the working configuration. To account for the parameter search, age-group labels were permuted 5000 times, and the maximum Ση^2^ across configurations was compared with its permutation distribution.

All analyses were exploratory. The significance level was set at 0.05; *p* values were not adjusted for the number of processing configurations, except in the permutation analysis described above.

## 3. Results

### 3.1. Study Cohort

The demographic characteristics of the cohort are presented in Table 1. The sex distribution did not differ significantly between groups (*p* = 0.117), although Group 3 included no men. Spherical equivalent did not differ significantly between groups (*p* = 0.134).

### 3.2. Effect of the Local Window Radius (RSWEEP Mode)

At the working configuration, Shapiro–Wilk and Levene’s tests did not indicate departures from normality or homogeneity of variance for VD, VesselArea, and VLD (*p* > 0.05). For IVD, normality was rejected in the young and older groups (Shapiro–Wilk *p* = 0.013 and 0.020), and variances were unequal (Levene’s *p* = 0.014); the IVD age-group difference was therefore also examined by Welch ANOVA and the Kruskal–Wallis test.

**Figure 4:**
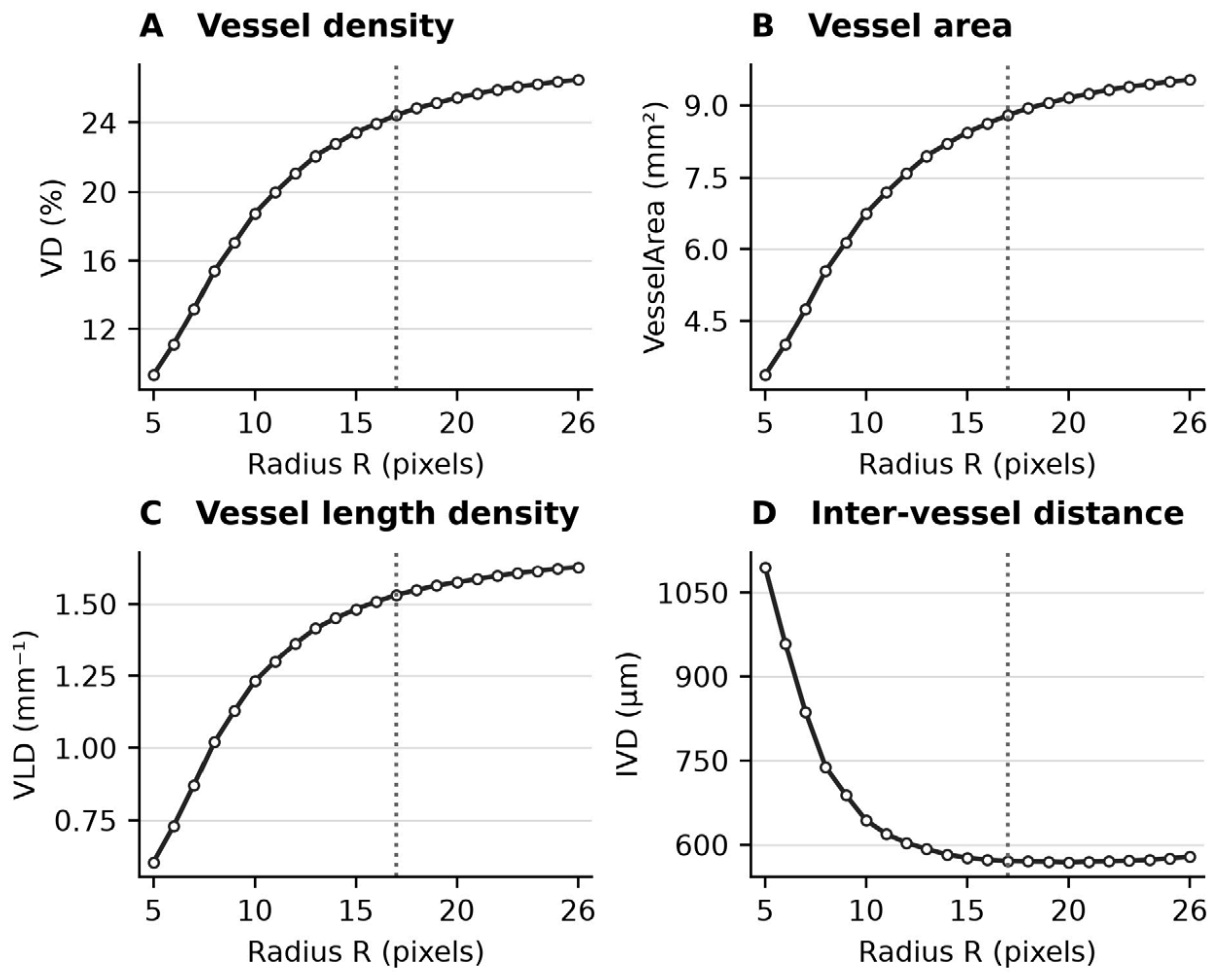
Dependence of cohort mean Haller layer parameter values on the local Phansalkar radius R (k = 0.25, r = 0.50; 66 eyes). Panels show (**A**) VD, (**B**) VesselArea, (**C**) VLD, and (**D**) IVD over R = 5–26 pixels. Each panel has its own labeled vertical scale. Dotted lines mark the representative working radius R = 17 pixels (approximately 319 µm).

As R increased from 5 to 26 pixels, overall mean VD rose from 9.34 to 26.50%, VesselArea from 3.36 to 9.54 mm^2^, and VLD from 0.60 to 1.63 mm^−1^, whereas mean IVD decreased markedly at smaller radii and then approached a flatter region (Figure 4). At R = 17 pixels, the age pattern was non-monotonic: VD, VesselArea, and VLD were highest in the middle-aged group and lowest in the older group (Table 2). Only the middle-aged versus older comparison remained significant after Bonferroni adjustment for the three group pairs (VD and VesselArea, *p* = 0.036; VLD, *p* = 0.034; IVD, *p* = 0.032), whereas differences between young and older participants were in the same direction but did not reach significance (*p* = 0.138–0.608). The older group differed from the middle-aged group by −3.68% in VD (Bonferroni-adjusted 95% CI −7.18 to −0.18), −0.20 mm^−1^ in VLD (−0.39 to −0.01), and +60.9 µm in IVD (4.0 to 117.9). Mean IVD was numerically higher in the older group (609.6 ± 104.5 µm) than in the young and middle-aged groups (561.5 ± 71.1 and 548.7 ± 49.0 µm, respectively), with an overall age-group difference by one-way ANOVA (*p* = 0.029; η^2^ = 0.106) that was not confirmed by Welch ANOVA (*p* = 0.071) or the Kruskal–Wallis test (*p* = 0.102).

Age-group effect sizes varied non-monotonically with R. Significant differences (*p* < 0.05) were obtained for VD, VesselArea, and IVD at R = 12–26 pixels and for VLD at R = 13 and R = 15–26 pixels. Maximum η^2^ occurred at R = 18 for VD and VesselArea (0.097), R = 19 for VLD (0.109), and R = 23 for IVD (0.133). At R = 17 pixels, η^2^ was 0.096 for VD and VesselArea (both *p* = 0.041), 0.104 for VLD (*p* = 0.031), and 0.106 for IVD (*p* = 0.029), whereas MeanDiameter and VesselCount did not differ between groups (*p* = 0.053 and 0.125, respectively). These effect sizes indicate modest group differences.

Ση^2^ reached a plateau at R = 13–26 pixels, where it varied little (0.446–0.491, within 10% of its maximum), and declined steeply at smaller radii (0.420 at R = 12 and 0.394 at R = 11). R = 17 pixels, whose Ση^2^ (0.459) was close to the plateau median, was used as a representative working radius in the krSWEEP analysis rather than as an optimal setting. A permutation test accounting for the 22 tested radii indicated that age-group separation exceeded chance (*p* = 0.031).

### 3.3. Effect of the Parameters k and r (krSWEEP Mode)

Four of the twenty-four configurations produced significant age-group differences for VD (and the equivalent VesselArea), VLD, and IVD simultaneously (Table 3).

Individually, VD/VesselArea reached *p* < 0.05 in these four configurations only, VLD in eight, and IVD in eleven. Maximum η^2^ occurred at k = 0.25, r = 0.50 for VD/VesselArea (0.096; *p* = 0.041), at k = 0.20, r = 0.50 for VLD (0.134; *p* = 0.011), and at k = 0.40, r = 0.25 for IVD (0.117; *p* = 0.020).

Ση^2^ was highest at k = 0.20, r = 0.50 (0.468) and k = 0.25, r = 0.50 (0.459) and ranged from 0.121 to 0.425 in the other configurations. Thus, three configurations met the plateau criterion (Ση^2^ ≥ 0.421). The difference between the two leading pairs was negligible (+0.009; 95% CI: −0.189 to 0.228). In the four-parameter sensitivity analysis, k = 0.25, r = 0.50 ranked first (Ση^2^ = 0.395). After accounting for the search across 24 k/r combinations, the age-group signal did not exceed chance (permutation *p* = 0.132; across all 45 configurations, *p* = 0.115). The pair k = 0.25, r = 0.50, corresponding to the default Phansalkar values, was chosen as the working configuration by convention.

### 3.4. Sensitivity Analyses

At the working configuration, VD, VLD, and IVD were strongly intercorrelated (|*r*| = 0.83–0.93). After adjustment for sex and spherical equivalent, the age-group differences remained significant for VD (*p* = 0.035), VLD (*p* = 0.042), and IVD (*p* = 0.044). With age as a continuous variable adjusted for sex and spherical equivalent, VLD changed by −0.047 mm^−1^ per decade (95% CI: −0.087 to −0.006; *p* = 0.025) and IVD by +13.5 µm per decade (95% CI: 1.4 to 25.7; *p* = 0.030), whereas the association for VD was not significant (−0.68% per decade; 95% CI: −1.42 to 0.06; *p* = 0.072). A quadratic age term was not significant for any of these parameters (*p* = 0.062–0.175). In women only (*n* = 58), the associations with age were preserved for VLD (*p* = 0.034) and IVD (*p* = 0.044).

The SCP exclusion mask covered 4.4 ± 1.9% of the field (range 0.6–9.2%); it did not differ significantly between age groups (5.1 ± 2.0%, 3.9 ± 1.8%, and 4.3 ± 1.9%; *p* = 0.091) and was not correlated with age (r = −0.17, *p* = 0.17). After normalization to the analyzable area, the age-group difference was preserved for VLD (*p* = 0.041; η^2^ = 0.097) but not for VD (*p* = 0.058; η^2^ = 0.086). Excluding mask-affected maxima reduced IVD by 36 ± 27 µm (85% of maxima retained; r = 0.94 with the original values); the age-group difference in IVD was no longer significant (*p* = 0.080), whereas its association with age in sex- and spherical equivalent-adjusted regression was preserved (+13.4 µm per decade; 95% CI: 2.3 to 24.5; *p* = 0.018), as was that of analyzable-area VLD (−0.053 mm^−1^ per decade; 95% CI: −0.097 to −0.009; *p* = 0.018).

## 4. Discussion

Phansalkar was selected by a qualitative comparison of nine local algorithms on images from 30 eyes (Section 2.3). This comparison supported its use for parameter evaluation but does not establish superior binarization accuracy.

Processing settings also differ across implementations: Lee et al. used Phansalkar binarization with a radius of 100 pixels for en face Haller images [15], whereas Valsecchi et al. applied it within 16 × 16-pixel windows on B-scans for three-dimensional reconstruction [12]. Comparability therefore requires reporting the image type, slab definition, native pixel scale, and threshold parameters rather than transferring a numerical radius between workflows.

We compared processing configurations by age-group differences, using the summed effect size across five parameters, excluding the redundant VesselArea, as the comparison criterion. At the working configuration, three parameters—VD (equivalent to VesselArea for a fixed field), VLD, and IVD—differed modestly between age groups by one-way ANOVA (η^2^ = 0.096–0.106), whereas MeanDiameter and VesselCount did not. Because these three parameters were strongly intercorrelated (|r| = 0.83–0.93), they provide largely overlapping rather than independent evidence. The purpose was to define an explicit working configuration, not to establish normative values, measurement repeatability, or clinical predictive performance. Because configurations were compared by age-group separation in the same cohort, this approach is partly circular: a configuration that maximizes group differences in 66 eyes is not necessarily the one that most accurately represents Haller vessels. The permutation analysis indicated that age-group separation exceeded chance across radii (*p* = 0.031) but not across all 45 configurations (*p* = 0.115); the nominal *p* values should therefore be interpreted as exploratory.

The local radius controls the spatial scale over which image statistics are calculated. VD, VesselArea, and VLD increased with R, whereas IVD fell markedly at small radii before approaching a flatter region. Effect-size maxima did not coincide (R = 18 for VD/VesselArea, R = 19 for VLD, and R = 23 for IVD), but Ση^2^ varied little over R = 13–26 pixels, and R = 17 lies within this plateau. The working radius of 17 pixels (approximately 319 µm) is therefore a representative value within this range rather than an optimal setting; it should not be equated with vessel diameter or regarded as a physiological or statistical optimum and requires verification before being applied to other images, devices, or datasets.

The parameters k and r modify the contribution of local contrast to the Phansalkar threshold [25], and their effects depend on local image statistics. The pairs k = 0.20, r = 0.50 and k = 0.25, r = 0.50 produced the highest and nearly identical summed effect sizes, but bootstrap confidence intervals for differences between configurations were wide; the latter, k = 0.25 and r = 0.50, corresponds to the original Phansalkar values, which are the defaults in Fiji; it was retained to allow reproduction without additional tuning.

The age pattern was not a progressive decline. VD (and the equivalent VesselArea) and VLD were highest in the middle-aged group, and the only pairwise difference that survived Bonferroni adjustment was between middle-aged and older participants; differences between young and older participants were in the same direction but not significant. When age was analyzed as a continuous variable with adjustment for sex and spherical equivalent, VLD decreased and IVD increased with age, whereas the association for VD did not reach significance. Likewise, after normalization to the analyzable area, the age-group difference was preserved for VLD (*p* = 0.041) but not for VD (*p* = 0.058), indicating that the nominal VD difference was sensitive to the choice of denominator; the age-related signal was therefore more consistent for VLD than for VD. Given the non-monotonic group means, these linear associations should be interpreted as an average trend across the age range rather than as evidence of a steady decline; the absence of a significant quadratic term in 66 eyes does not exclude a non-linear pattern. The lower values in the older group are consistent in direction with reported age-associated reductions in choroidal vascular measures on en face images, structural B-scans, and three-dimensional models [6,26,27,28], but the present data do not establish these parameters as progressively age-related. These associations should also be interpreted with caution because the oldest group included no men and axial length was not measured, although the associations for VLD and IVD were preserved in women only.

IVD complements density-based measurements by describing the spaces between segmented vessels. Its greater mean in the older group should be interpreted with caution: the age-group difference was significant by one-way ANOVA (*p* = 0.029) but was not confirmed by Welch ANOVA (*p* = 0.071) or the Kruskal–Wallis test (*p* = 0.102), which were applied because IVD was not normally distributed and was most variable in the older group (SD 104.5 versus 71.1 and 49.0 µm), and it was not robust to the exclusion of masked regions (*p* = 0.080). IVD remained associated with age as a continuous variable, but given this dispersion, the association should also be regarded as exploratory; this study does not establish its clinical superiority over the other metrics. Unlike manual measurements between selected vessels [13], the present IVD is derived from distance-map maxima over the binary background and is altered by thresholding and SCP exclusion; it should therefore be interpreted as an image-derived measure rather than a direct anatomical distance.

The binary masks were fragmented, with vessels often represented by short segments and small compact islands (Figure 3A). Morphological opening with a square kernel, local-thickness filtering, and subtraction of the SCP exclusion mask can each interrupt vessels; in addition, a finite en face slab intersects vessels that run obliquely through its boundaries. Fragmentation also affects skeleton-based measures: gaps shorten the skeleton, whereas small islands and irregular contours add short branches. VesselCount and VLD should therefore be interpreted as properties of the binary mask rather than as direct anatomical counts or lengths.

This exploratory pilot study has several limitations. Processing parameters were selected and evaluated in the same 66 eyes, and no independent validation cohort was available. The comparison criterion included strongly intercorrelated parameters. The oldest group included no men, and axial length was not measured. Sex and spherical equivalent were included as covariates, but this adjustment could not fully separate the effects of sex and age, spherical equivalent is not a substitute for axial length, and residual confounding of the age-related associations cannot be excluded [21,22,23,24]. Manual slab placement was performed by a single masked grader, and neither its reproducibility nor the test–retest repeatability of the measurements was assessed. No manually annotated reference standard was available, and the algorithm comparison was qualitative, unmasked to algorithm identity, and performed before FFT filtering. All data were obtained with one device at a single center.

## 5. Conclusions

Quantitative measurements of the Haller layer on structural en face OCT images depend substantially on postprocessing parameters; image scale and threshold settings should therefore be reported together with the results. For 320 × 320-pixel images covering 6 × 6 mm, R = 17 pixels (approximately 319 µm), k = 0.25, and r = 0.50 provide an explicitly defined working configuration; R = 17 pixels is a representative value within a broad range of radii (R = 13–26 pixels) with similar age-group separation, not an optimal setting. After accounting for the parameter search, age-group separation exceeded chance across the 22 tested radii (permutation *p* = 0.031) but not across all 45 configurations (*p* = 0.115); its robustness to the full parameter search is therefore not established. With the working configuration, the age-group difference in VLD remained nominally significant after normalization to the analyzable area (*p* = 0.041), whereas that in VD did not (*p* = 0.058). The age-group difference in IVD was not confirmed by Welch ANOVA or the Kruskal–Wallis test, given its greater variability in the older group, and was not significant after exclusion of masked regions. After adjustment for sex and spherical equivalent, VLD and IVD showed modest associations with age as a continuous variable, also after accounting for the SCP exclusion mask, whereas VD did not; these associations cannot be fully separated from sex- and axial length-related effects, as the oldest group included no men and axial length was not measured. Because the parameters were selected and evaluated in the same cohort, these findings are exploratory; test–retest repeatability and validation in an independent cohort are required before these measurements are used as quantitative markers.

## Figures and Tables

**Table 1:** Demographic characteristics of the cohort (66 volunteers, 66 eyes).

Parameter	Group 1 (22–40 Years)	Group 2 (41–60 Years)	Group 3 (61–73 Years)	*p*
Volunteers, *n*	22	24	20	–
Eyes, *n*	22	24	20	–
Age, years	28.6 ± 5.6	51.7 ± 6.9	67.8 ± 3.0	<0.001 *
Sex (male/female), *n*	4/18	4/20	0/20	0.117 †
Spherical equivalent, D	−1.09 ± 1.44 (−3.0 to 0.00)	−0.32 ± 1.69 (−3.0 to +2.00)	−0.05 ± 2.03 (−3.0 to +3.00)	0.134

Values are *n* or mean ± standard deviation; spherical equivalent is given as mean ± standard deviation (range). Group 1: 22–40 years; Group 2: 41–60 years; Group 3: 61–73 years. *n*, number; D, diopters. *p* for age and spherical equivalent is from one-way ANOVA. * Statistically significant difference (*p* < 0.05). † Fisher’s exact test (Freeman–Halton extension).

**Table 2:** Quantitative parameters of the Haller layer by age group at R = 17 pixels (approximately 319 µm), k = 0.25, and r = 0.50 (RSWEEP).

Parameter	Group 1 (22–40 Years)	Group 2 (41–60 Years)	Group 3 (61–73 Years)	*p*	η^2^ (95% CI)
VD, %	24.34 ± 3.77	26.15 ± 5.48	22.47 ± 4.60	0.041 *	0.096 (0.012–0.282)
VesselArea, mm^2^	8.76 ± 1.36	9.41 ± 1.97	8.09 ± 1.66	0.041 *	0.096 (0.012–0.282)
VLD, mm^−1^	1.56 ± 0.24	1.61 ± 0.26	1.41 ± 0.27	0.031 *	0.104 (0.018–0.289)
IVD, µm	561.5 ± 71.1	548.7 ± 49.0	609.6 ± 104.5	0.029 *	0.106 (0.018–0.280)

Values are mean ± standard deviation; *p* and η^2^ are from one-way ANOVA; 95% CIs for η^2^ were obtained by age-stratified bootstrap (5000 replicates). Group 1: 22–40 years (*n* = 22); Group 2: 41–60 years (*n* = 24); Group 3: 61–73 years (*n* = 20). VD, vessel density; VesselArea, vessel area; VLD, vessel length density; IVD, inter-vessel distance; CI, confidence interval. For the fixed 36 mm^2^ field, VesselArea is a linear transformation of VD (VesselArea = 0.36 × VD) and is shown for completeness. * *p* < 0.05.

**Table 3:** Age-group effect sizes (η^2^) for Haller layer parameters at different combinations of k and r and a fixed radius R = 17 pixels (approximately 319 µm; krSWEEP mode).

k	r	VD (or VesselArea)	VLD	IVD
0.20	0.50	0.091 *	0.134 *	0.104 *
0.20	0.75	0.092 *	0.120 *	0.114 *
0.25	0.50	0.096 *	0.104 *	0.106 *
0.40	0.25	0.092 *	0.102 *	0.117 *

The four of 24 configurations with significant age-group differences (*p* < 0.05) for all three parameters are shown. Values are η^2^ from one-way ANOVA. VD, vessel density; VesselArea, vessel area; VLD, vessel length density; IVD, inter-vessel distance. * *p* < 0.05.

## Data Availability

The de-identified data and image-processing macro supporting the findings of this study are available from the corresponding author upon reasonable request.

## Abbreviations

ANOVA	analysis of variance
CI	confidence interval
FFT	fast Fourier transform
Fiji	Fiji Is Just ImageJ
IVD	inter-vessel distance (distance-map-derived intervascular space diameter)
krSWEEP	systematic variation of the Phansalkar k and r parameters
MeanDiameter	mean vessel diameter
OCT	optical coherence tomography
OCTA	optical coherence tomography angiography
PAR	projection artifact removal
RSWEEP	systematic variation of the Phansalkar window radius
SCP	superficial capillary plexus
SD	standard deviation
TIFF	Tagged Image File Format
VD	vessel density
VesselArea	vessel area
VesselCount	number of connected binary objects
VLD	vessel length density
